# APPA (apocynin and paeonol) modulates pathological aspects of human neutrophil function, without supressing antimicrobial ability, and inhibits TNFα expression and signalling

**DOI:** 10.1007/s10787-020-00715-5

**Published:** 2020-05-07

**Authors:** A. L. Cross, J. Hawkes, H. L. Wright, R. J. Moots, S. W. Edwards

**Affiliations:** 1 Institute of Ageing and Chronic Disease, Aintree University Hospital, Longmoor Lane, Liverpool, L9 7AL UK; 2 Institute of Integrative Biology, University of Liverpool, Liverpool, L69 7ZB UK; 3 Department of Biochemistry, Institute of Integrative Biology, University of Liverpool, Liverpool, L69 7ZB UK

**Keywords:** Neutrophil, APPA, Paeonol, Apocynin, NFκB, Rheumatoid arthritis

## Abstract

Neutrophils are key players in the pathophysiological process underlying inflammatory conditions not only by release of tissue-damaging cytotoxic enzymes, reactive oxygen species (ROS) but also by secretion of important immunomodulatory chemokines and cytokines. Here, we report the effects of the novel agent APPA, undergoing formal clinical development for treatment of osteoarthritis, and its constituent components, apocynin (AP) and paeonol (PA) on a number of neutrophil functions, including effects on TNFα- expression and signalling. Neutrophils were treated with APPA (10–1000 µg/mL) prior to the measurement of cell functions, including ROS production, chemotaxis, apoptosis and surface receptor expression. Expression levels of several key genes and proteins were measured after incubation with APPA and the chromatin re-modelling agent, R848. APPA did not significantly affect phagocytosis, bacterial killing or expression of surface receptors, while chemotactic migration was affected only at the highest concentrations. However, APPA down-regulated neutrophil degranulation and ROS levels, and decreased the formation of neutrophil extracellular traps. APPA also decreased cytokine-stimulated gene expression, inhibiting both TNFα- and GM-CSF-induced cell signalling. APPA was as effective as infliximab in down-regulating chemokine and IL-6 expression following incubation with R848. Whilst APPA does not interfere with neutrophil host defence against infections, it does inhibit neutrophil degranulation, and cytokine-driven signalling pathways (e.g. autocrine signalling and NF-κB activation), processes that are associated with inflammation. These observations may explain the mechanisms by which APPA exerts anti-inflammatory effects and suggests a potential therapeutic role in inflammatory diseases in which neutrophils and TNFα signalling are important in pathology, such as rheumatoid arthritis.

## Introduction

Advances in identifying the pathophysiological processes underlying systemic inflammatory disease such as rheumatoid arthritis (RA) have led to the development of targeted therapies and enhanced outcomes for many patients (Nikiphorou et al. 2017). However, no single drug is effective for all patients and each is associated with significant risks of adverse effects demonstrating a need for novel, safe and effective therapies. A greater understanding of neutrophil biology has led to an appreciation that these cells play a significant role in many systemic inflammatory including RA (Wright et al. 2014a, b), but targeting neutrophils therapeutically has proven most challenging, as host defence must not be compromised. Neutrophils contribute to inflammatory diseases via the release of cytokines, chemokines, reactive oxygen species (ROS) and proteases (Jaillon et al. 2013) that are activated via distinct but sometimes overlapping agonist:signal transduction pathways. Successful targeting of neutrophils as therapy in inflammatory diseases must therefore block tissue-damaging processes (e.g. secretion) but not interfere with opsono-phagocytosis or microbial killing.

TNFα is a key molecule in the pathology of many autoimmune conitions such as RA and therapeutic targeting of this molecule, e.g. with biologics such as TNFi, can lead to dramatic improvements in many, but not all patients (Hyrich and Watson 2006; Emery et al. 2014). In RA, neutrophils contribute to abnormal TNFα signalling by both responding to and expressing this cytokine during active disease (Wright et al. 2010). Membrane bound (m)TNFα is elevated on the surface of blood neutrophils in RA patients with active disease, and NF-κB activation levels (which may be triggered via TNFα signalling) are elevated, but both surface mTNFα levels and NF-κB activation levels return to healthy control levels during successful TNFi therapy (Wright et al. 2010). NF-κB is a key regulatory protein involved in inflammatory processes in a wide range of conditions from RA to osteoarthritis (OA) (Pilichou et al. 2008). It regulates the functions of all cell types involved in joint physiology and pathology, including synoviocytes, chondrocytes, osteocytes, endothelial cells, vascular smooth muscle cells, fibroblasts and leukocytes (Bonizzi and Karin 2004), and so targeting its over-activity may be beneficial in such diseases (Gilmore and Herscovitch 2006). This transcription factor plays a central role in the regulation of a number of neutrophil functions, and it is constitutively activated in many patients with active RA (Muller-Ladner et al. 2002), likely via TNFα signalling (Kanbe et al. 2008). More recently, a new mechanism of gene activation in neutrophils involving either endogenously expressed or exogenously added TNFα has been identified following incubation of neutrophils with the TLR8 agonist/chromatin remodelling agent, R848 (Zimmermann et al. 2015). Human neutrophils do not normally express IL-6 because the promoter region of this gene is transcriptionally “silent” and in an inactive configuration. However, R848 alters chromatin structure at this locus to enable its transcription. Both endogenously expressed and exogenously added TNFα are required for this IL-6 expression by prolonging the synthesis of IκBζ co-activator and sustaining C/EBPβ recruitment and histone acetylation at *IL-6* regulatory regions (Zimmermann et al. 2015). In view of the importance of IL-6 and TNFα in the pathology of RA, this mechanism of endogenously expressed TNFα on expression of IL-6 on re-modelled chromatin could be extremely important in understanding disease mechanisms. Targetting these processes could, therefore, have significant therapeutic benefits.

APPA, a synthetic combination of two anti-inflammatory molecules, apocynin (AP) and paeonol (PA), has shown efficacy in canine models of OA (Glasson and Larkins 2012; Larkins and King 2017a, b) and is currently under clinical development for use in human OA. Its efficacy is thought to lie predominantly in its effects on regulation of the transcription factor, NF-κB as well as other signalling pathways (Muller-Ladner et al. 2002). AP is a strong ROS scavenger (Nam et al. 2014; Stefanska and Pawliczak 2008; Impellizzeri et al. 2011a, b) and inhibits the expression and release of several inflammatory cytokines and matrix metalloproteinases; while PA, an isomer of apocynin, down-regulates activation, nuclear translocation, and DNA binding of NF-κB (Su et al. 2010). These combined activities of APPA inhibit many of the molecular events triggered during inflammatory activation. However, the effects of APPA and its constituent components on neutrophil function, many of which are regulated by TNFα, are completely unknown. Given the proposed mechanisms of action of this drug, it might be predicted to down-regulate inflammatory responses in neutrophils that are regulated by NF-κB. The aims of this research were to investigate the effects of APPA, PA and AP on neutrophils in vitro, especially on functions that regulate host defence against infections. We also investigated the ability of these molecules to modulate R848-induced IL-6 expression via inhibition of endogenous TNFα activity and show that it is as effective as TNFα-blocking antibodies in this action, yet does not have any observable inhibition on neutrophil host defence.

## Materials and methods

### Isolation of neutrophils

Blood was collected into lithium-heparin vacutainers from healthy controls, after giving informed consent: this study was approved by the NHS Health Research Authority (Inflammatory Signalling Pathways; Ref 11/NW/0206: IRAS project ID 75388). Neutrophils were isolated following sedimentation in HetaSep and centrifugation on Ficoll-paque (Wright et al. 2016): contaminating erythrocytes were removed by hypotonic lysis. Neutrophils were examined for purity by Romanowsky staining and microscopic analysis of cytospins, and viability by trypan blue exclusion; these were > 97% and > 98%, respectively in freshly isolated cells. Neutrophils were incubated at 10^6^ or 5 × 10^6^ cells/mL (as described in the text) in RPMI media (Thermo-Fisher) plus 10% human AB serum (Sigma) and incubated at 37 °C and 5% CO_2_ for up to 20 h. Cytokines were added as follows: IL-8 (100 ng/mL, Sigma); GM-CSF (5 ng/mL, Roche); TNFα (10 ng/mL, Merck); IL-1β (10 ng/mL, Source Bioscience); IFNγ (10 ng/mL, Source Bioscience). R848 (Sigma) was used at a concentration of 5 µM (Zimmermann et al. 2015). APPA (a 2:7 ratio of AP:PA) was dissolved in DMSO and was initially tested over a concentration range of 10–1000 µg/mL (final concs). AP and PA were also used individually at the concentrations equivalent in the APPA mixture used at 100 µg/mL.

### Measurement of apoptosis

Neutrophils (1 × 10^5^) were removed from culture (at the indicated times), diluted with 100 μL of HBSS (Thermo-Fisher) containing 0.5-μL annexin V-FITC (Thermo-Fisher), and incubated in the dark at room temperature for 15 min. The total volume was then made up to 500 μL with HBSS, and propidium iodide added (final concentration 1 μg/mL, Sigma) before analysis immediately on a Dako Cyan ADP flow cytometer. 10,000 events/sample were analysed.

### Degranulation

Neutrophils (5 × 10^6^/mL) were pre-incubated for 10 min with APPA (100 µg/mL), before priming with GM-CSF (5 ng/mL) for 30 min and then stimulated to degranulate with cytochalasin B (5 µg/mL) plus fMLP (1 µM, both from Sigma) for 15 min. Cells were pelleted gently, washed and analysed by flow cytometry; while, supernatants were removed for SDS-PAGE after adding concentrated Laemmli protein sample buffer. After electrophoresis, proteins were transferred to PVDF membranes and probed with antibodies to myeloperoxidase (MPO) (R&D Systems), lactoferrin (Abcam), MMP9 (R&D Systems) and elastase (Abcam). Secondary antibodies were anti-rabbit IgG (GE Healthcare) and anti-mouse IgG (Sigma) HRP-linked antibodies (1:10,000). Bound antibodies were detected using the ECL system (Merck) and film (Amersham).

### Antibody staining and flow cytometry

Antibody staining was carried out on freshly isolated neutrophils incubated for up to 1 h, as described above. Neutrophils (1 × 10^5^) were resuspended in PBS (+ 0.2% BSA). Antibody binding was carried out at 4 °C in the dark for 30 min with conjugated antibodies added as follows: CD62L-FITC (R&D systems); CD11b-PE (R&D systems); CD16-PE (R&D systems); CD18-PE (R&D systems); CD63-APC (Thermo-Fisher); CD64-FITC (R&D systems); IL-8R (CXCR1)-FITC (R&D systems); CD66b-FITC (R&D systems); and isotype controls (Santa Cruz). Fluorescence was measured immediately on a Dako Cyan ADP flow cytometer. 10,000 events/sample were analysed.

### Western blotting

Proteins from 5 × 10^5^ cells, extracted in Laemelli buffer containing protease- and phosphatase inhibitors, were separated by SDS-PAGE using a 12% gel and transferred onto PVDF membranes (Merck). Primary antibodies were: NF-κB (1:1000, Cell Signaling); IκBα (1:1000, Cell Signaling); Erk1/2 (1:1000, Cell Signaling); p38-MAPK (1:1000, Cell Signaling) and GAPDH (1:10,000, Abcam). Secondary antibodies were anti-rabbit IgG (GE Healthcare) and anti-mouse IgG (Sigma) HRP-linked antibodies (1:10,000). Bound antibodies were detected using the ECL system (Millipore) on carefully exposed film (Merck) to avoid saturation.

### Chemotaxis assay

Chemotaxis was performed in 24-well tissue culture plates (coated with 12 mg/mL poly-hema (Sigma)) using hanging cell inserts (Merck) with a 3-μm-pore membrane separating media in the upper and lower chambers. Standard neutrophil chemotactic agents were added to 800-µL RPMI media in the lower chamber (fMLP at 10^−8^ M and IL-8 at 100 ng/mL, final concs, both from Sigma). Neutrophils (10^6^) were added to the upper chamber and the plates incubated for 90 min at 37 °C and 5% CO_2_. The number of migrated neutrophils in the lower chamber after 90 min was measured using a Coulter Counter Multisizer3 (Beckman Coulter).

### Respiratory burst measurements

Neutrophils (5 × 10^6^/mL) were pre-incubated for 10 min with APPA (10–1000 µg/mL), before incubation with GM-CSF (5 ng/mL) or TNFα (10 ng/mL) for 30 min. Cells (5 × 10^5^) were then added to wells of a 96-well plate and diluted in HBSS containing luminol (10 μM) and the respiratory burst stimulated with fMLP (1 μM, Sigma) or PMA (100 ng/mL, Sigma). Luminescence was measured using a Tecan GENios Plus Luminescence plate reader measuring continuously for 30 min.

### Opsonisation and phagocytosis of bacteria

*Staphylococcus aureus* (Oxford strain) were heat-killed by incubation at 60 °C for 30 min, washed twice, and then resuspended in PBS containing 30-µM propidium iodide (PI). The suspension was incubated in the dark at 4 °C for 2 h and then washed. Opsonisation, using pooled human AB serum from healthy donors (stored in aliquots at − 20 °C), was achieved by incubating bacteria (5 × 10^8^/mL) with 10% heat-inactivated human serum (*v*/*v*, final concentration) for 30 min at 37 °C before washing. Freshly isolated neutrophils (10^6^/mL) from healthy controls were incubated for 30 min with PI-labelled, opsonised heat-killed *S. aureus* (SAPI) in a ratio of 1:10 and incubated in the dark for 30 min at 37 °C with gentle agitation. Neutrophils were then pelleted by centrifugation, washed twice, and suspended in PBS containing 5-mM EDTA, 3-mM sodium azide and 1% paraformaldehyde followed by analysis using flow cytometry.

### Bacterial killing

Freshly grown *S. aureus* were harvested and washed, and suspended at 5 × 10^8^/mL in HBSS and opsonised as described above. Freshly isolated neutrophils (10^6^/mL) were incubated for 1 h at 37 °C with gentle agitation with opsonised bacteria at a ratio of 1:10. Neutrophils were then lysed to release live bacteria by serial dilution in distilled water and vigorous vortexing, before being plated onto LB agar plates and incubated overnight. Colonies were counted and results calculated as percentage of bacteria killed compared to bacteria only (no neutrophils) samples.

### NET formation

(a) Quantitation of DNA release: Neutrophils (5 × 10^5^/500 µL media containing 2% (*v*/*v*) FBS) were seeded into wells of a 24-well culture and incubated for 1 h at 37 °C. APPA (100 µg/mL) was then added and incubated for 10 min before stimulation with 100nM PMA for 3 h at 37 °C. After incubation, NET DNA was isolated using Micrococcal nuclease (500 mU, Sigma) and quantified utilizing picogreen (Promega) and a DNA calibration curve. (b) microscopic visualisation: neutrophils were seeded and incubated as described above. Following incubation cells were fixed on cover slips, stained with neutrophil elastase antibody and DAPI (Thermo-Fisher) before being viewed microscopically on a Leica TCS SPE (Papayannopoulos et al. 2010).

### Gene expression using qPCR

1 × 10^7^ neutrophils (5 × 10^6^/mL) were pre-incubated for 10 min with APPA (10–1000 µg/mL), before incubation with GM-CSF (5 ng/mL), TNFα (10 ng/mL) or IFNγ (10 ng/mL) alone or in combination for 1 h. Cells were then immediately pelleted and RNA extracted using Trizol (Thermo-Fisher) and stored at − 20 °C. RNA was cleaned with RNeasy kit (which included a DNAse step, Qiagen) before cDNA synthesis, which was amplified using primers for: TNFα (forward CAGAGGGCCTGTACCTCATC, reverse GGAAGACCCCTCCCAGATAG); CCL3 (forward GCTCTCTGCAACCAGTTCTCT, reverse TGGCTGCTCGTCTCAAAGTAG) AND CCL4 (forward GCTGTGGTATTCCAAACCAAAAGAA, reverse AGGTGACCTTCCCTGAAGACT). IL-6 was amplified using a Bio-Rad pre-validated primer pair. GAPDH was used to normalise samples (forward CTCAACGACCACTTTGTCAAGCTCA, reverse GGTCTTACTCCTTGGAGGCCATGTG). Results were quantified by the Pfaffl method and are expressed as fold increase/decrease compared to untreated neutrophils.

### Statistical analysis

Statistical analysis was carried out using SPSS v24, using Student’s *t* test unless otherwise stated.

## Results

### Apoptosis is accelerated by high concentrations of APPA

**Fig. 1:**
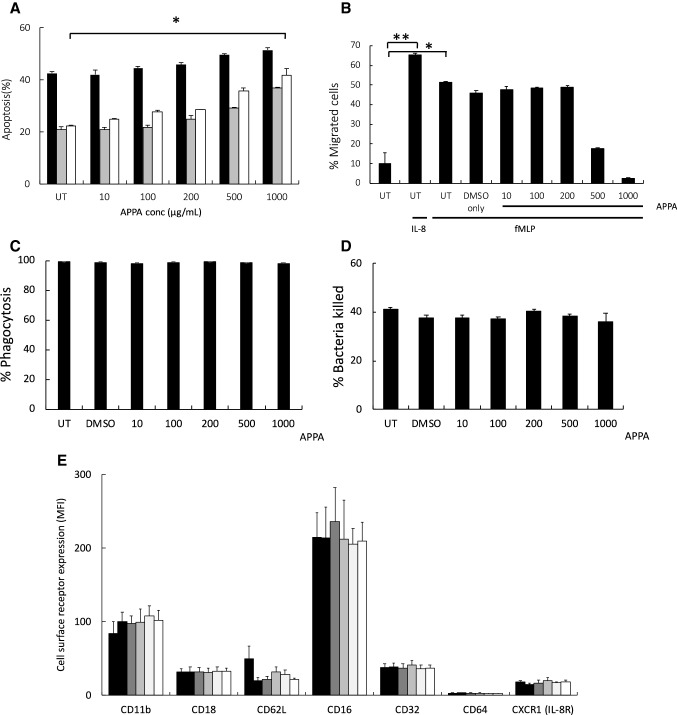
Effects of APPA on neutrophil apoptosis, chemotaxis, phagocytosis/killing and receptor expression. In **a** neutrophils (10^6^/mL) from healthy controls were incubated for 20 h in the absence (UT) or presence of APPA (10–1000 μg/mL) in the absence (control  ) or presence of cytokines known to regulate neutrophil apoptosis. Following 10-min pre-incubation with APPA, the following additions were made: GM-CSF (5 ng/mL,  ) or TNFα (10 ng/mL,  ) and incubation was continued for a further 20 h (n = 7). In **b** neutrophils (10^6^) from healthy controls were incubated in the absence (UT) or presence of APPA (10–1000 µg/mL) for 10 min, then migration towards fMLP (10^−8^ M) or IL-8 (100 ng/mL) was measured after a 90-min incubation period. Untreated neutrophils migrating towards fMLP (10^−8^ M) and IL-8 (100 ng/mL) are shown as positive controls (**p < 0.01, *p < 0.05). Values shown are means (± SEM, *n* = 4). In **c** and **d**, neutrophils were pre-incubated for 10 min with the indicated concentration of APPA (or DMSO vehicle control). In **c**, they were then incubated with a 10:1 ratio of PI-stained, heat-killed serum-opsonised *S. aureus* and phagocytosis was determined by flow cytometery. Values shown are mean MFI values (normalised to untreated control values of 100%), ± SD (*n* = 3). In **d**, neutrophils subsequently incubated with a 10:1 ratio of live, serum-opsonised *S.aureus* and after 1-h incubation, bacterial viability was determined by plate counting. Values shown are mean values ± SD (*n* = 3). In **e**, neutrophils were isolated from healthy controls and expression of cell surface receptors was measured on freshly isolated cells by flow cytometry. These levels of expression were compared with those on neutrophils pre-incubated with APPA (100 µg/mL) and stimulated for 1 h with either GM-CSF (5 ng/mL) or TNFα (10 ng/mL), as follows:  No additions;  TNFα only;  GM-CSF only;  APPA only;  TNFα + APPA;  GM-CSF + APPA. Levels of CD11b, CD18, CD16, CD32, CD64 and CXCR1 (IL-8R) were measured. Inset shows effects of APPA with and without GM-CSF or TNFα on CD62L expression levels. There was no significant difference in surface marker expression following treatment with APPA. Values shown are means ± SD (*n* = 3)

In initial experiments, neutrophils were pre-incubated with APPA (10–1000 µg/mL) in the presence or absence (control) of anti-apoptotic cytokines (GM-CSF or TNFα) for 20 h. While both GM-CSF and TNFα delayed neutrophils apoptosis (as described previously (Wright et al. 2010, 2014a, b; Moulding et al. 2001) levels of apoptosis in APPA-treated cells at 20 h were slightly increased above untreated (UT) control cells and while this effect was dose dependent, these effects did not reach statistical significance (Fig. 1a). APPA had a greater effect on apoptosis of cytokine-treated neutrophils, and at the highest concentration used significantly inhibited GM-CSF- and TNFα-delayed apoptosis (*p* < 0.01; Fig. 1a).

### Neutrophil chemotaxis is impaired at high APPA concentrations

Both IL-8 and fMLP are strong neutrophil chemottractants (Fig. 1b **p < 0.01, *p < 0.05, respectively, compared to no stimulus) and were used as positive controls to test the effects of APPA. Neutrophils were pre-incubated with APPA (10–1000 µg/mL) for 10 min, before measurement of chemotaxis for 90 min toward IL-8 (100 ng/mL, data not shown) or fMLP (0.01 µM, Fig. 1b). Chemotaxis towards both IL-8 (not shown) and fMLP in APPA-treated neutrophils was only inhibited at high concentrations of APPA (500 and 1000 µg/mL).

### APPA does not affect the ability of neutrophils to phagocytose and kill bacteria

Phagocytosis of PI-stained, serum-opsonised *S. aureus* was largely unaffected (> 96% phagocytosis, compared to untreated control values) by treatment with APPA at all concentrations used (Fig. 1c). Similarly, pre-incubation with APPA (10–1000 µg/mL) for 10 min did not impair killing of live, serum-opsonised *S. aureus*, with neutrophils killing ~ 40% bacteria over a 60-min incubation period at all concentrations tested, which was not significantly different from untreated controls (Fig. 1d).

### APPA does not alter surface receptor expression by neutrophils

Next, we examined the effect of APPA on expression of receptors that are important in chemotaxis and phagocytosis. Surface receptor expression of freshly isolated neutrophils, and neutrophils incubated for 1 h with GM-CSF and TNFα ± APPA (100 µg/mL) was measured by flow cytometry. As previously reported, GM-CSF (Fossati et al. 1998) and TNFα (Lynn et al. 1991) resulted in small increases in expression of CD11b (Fig. 1e), but APPA did not affect this up-regulation. Surface levels of CD18, CD16, CD32, CD64, l-selectin (CD62L) and CXCR1 (the IL-8 receptor) were unaffected by incubation with APPA (Fig. 1e).

### Effects of APPA on the respiratory burst

**Fig. 2:**
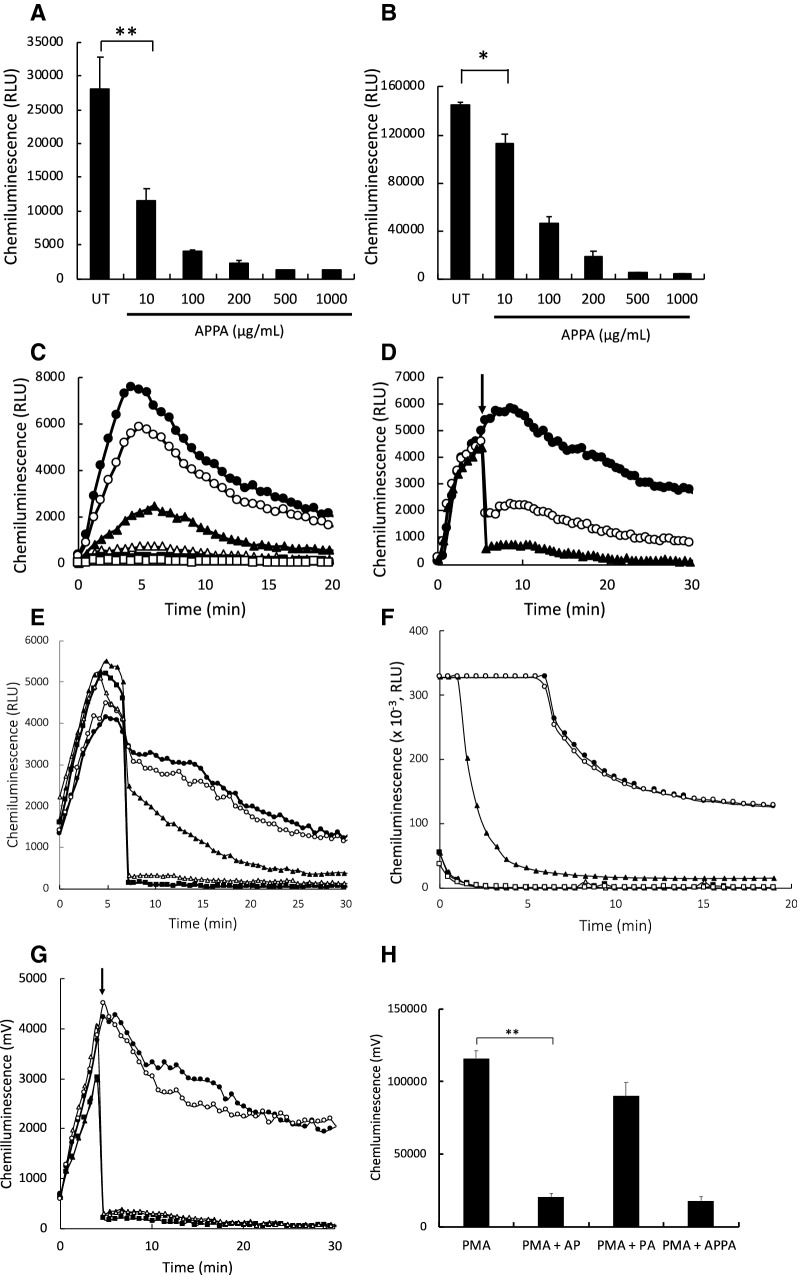
APPA decreases ROS production by activated neutrophils. Neutrophils (5 × 10^6^) from healthy controls were incubated in the absence (UT) or presence of APPA (10–1000 µg/mL) for 10 min perior to mesurements of luminol-enhanced chemiluminescence. In **a**, APPA-treated neutrophils were then primed for 30 min with 5 ng/mL GM-CSF before stimulating with fMLP (1 µM), *n* = 3, **p < 0.01; while in **b**, APPA-treated neutrophils were stimulated using PMA (100 ng/mL), *n* = 3, *p < 0.01. **c** Shows representative chemiluminescence traces of PMA-stimulated respiratory burst activity in the absence and presence of increasing concentrations of APPA: ( ) untreated controls, while  ,  ,  ,  ,  , show APPA concentrations of 10, 10, 200, 500 and 1000 µg/mL, respectively. In D PMA-induced respiratory burst activity was stimulated ( ) and after 5-min incubation, APPA (at 10 µg/mL,  and 100 µg/mL:  ) was added as indicated by the arrow. In **e** PMA was used to stimulate ROS production by neutrophils. As indicated by the arrow, the following additions were injected into the cell suspension:  , no additions;  , catalase (2U/mL);  , superoxide dismutase (40 µg/mL);  , sodium azide (1 mM);  , APPA (100 µg/mL). In **f** APPA (10–1000 µg/mL) or DMSO (as solvent control) were added to a cell-free luminol system utilizing hydrogen peroxide, as follows:  , no additions;  , DMSO;  , 10 µg/mL APPA;  , 100 µg/mL APPA;  , 200 µg/mL APPA;  , 500 µg/mL APPA. representative result of 3 separate experiments. In **g**, Neutrophils were stimulated with with PMA ( ) and after 5-min incubation APPA (100 µg/mL,  ), AP (22 µg/mL,  ) or PA (78 µg/mL,  ) added, as indicated by the arrow. **h** Shows replicate data of total chemiluminescence from **g**, **p value < 0.01, n = 11

Neutrophils were treated for 10 min with APPA (10–1000 µg/mL), before priming with GM-CSF (5 ng/mL) for 30 min. The respiratory burst was then stimulated via receptor-dependent or receptor-independent mechanisms with either fMLP (1 µM) or PMA (100 ng/mL), respectively. APPA decreased both the the fMLP-stimulated (Fig. 2a) and PMA-stimulated ROS levels (Fig. 2b,c) in a dose-dependent manner, with statistically significant inhibition evident at 10 µg/mL. As AP (a constituent of APPA) is a reported scavenger of ROS, we then added APPA 5 min after activation of the respiratory burst had been stimulated by PMA. Both concentrations of APPA used (10 µg/mL and 100 µg/mL) resulted in an immediate decrease in the chemiluminescence signal, as would be expected following addition of a ROS scavenging agent (Fig. 2d). For example, the addition of sodium azide (an inhibitor of myeloperoxidase) decreased ROS levels as rapidly as APPA; whereas, the addition of superoxide dismutase (which catalyses the conversion of O_2_^−^ into H_2_O_2_ and O_2_) resulted in a much slower decline in ROS levels in this experimental system (Fig. 2e). Further experiments utilising known reactive oxidant scavengers in a cell-free system (Fig. 2f) confirmed that ROS quenching/scavenging was largely responsible for the decrease in chemiluminescence signal by APPA. We then examined the effects of the individual components of APPA, namely AP and PA for their effects on neutrophil reactive oxidant scavenging. When these components were added 5 min after stimulation of the respiratory burst by PMA, PA had little effect on levels of reactive oxidants, whereas AP addition resulted in rapid and extensive quenching (*p* < 0.01), that was equivalent to the quenching effect seen by APPA (Fig. 2g, h).

### Effects of APPA on degranulation and NET formation

**Fig. 3:**
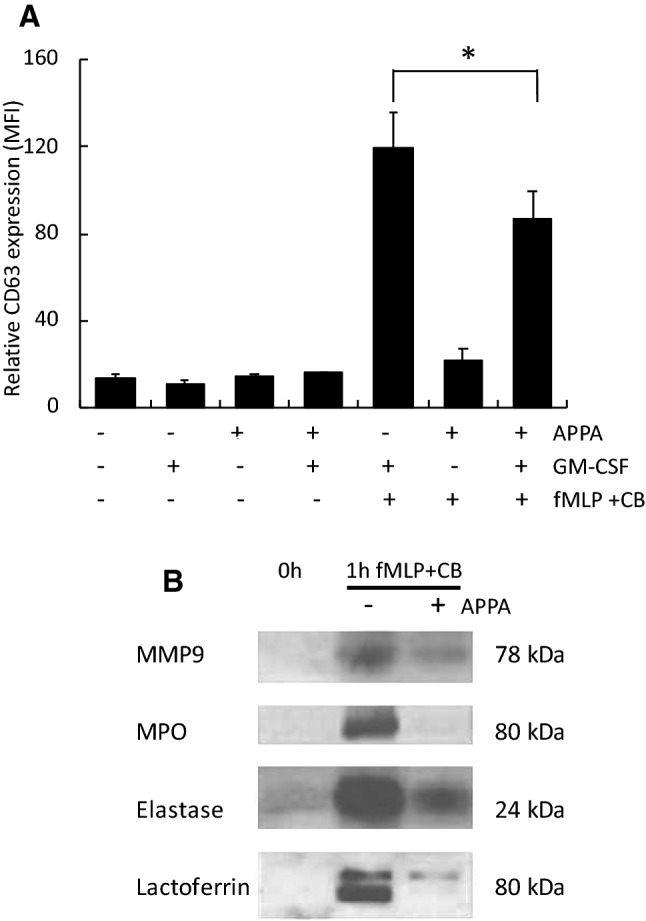
APPA decreases neutrophil degranulation. In **a** and **b**, neutrophils (5 × 10^6^) from healthy controls were incubated in the absence (UT) or presence of APPA (100 µg/mL) for 10 min. APPA-treated neutrophils were then primed for 30 min with GM-CSF before stimulating degranulation with fMLP (1 µM) plus cytochalasin B (5 µg/mL). In **a**, neutrophils were analysed for expression of CD63, a marker of degranulation using flow cytometry (*p < 0.05, *n* = 7). In **b**, supernatants from above were collected, proteins separated using SDS-PAGE before western blotting and probed for expression of MMP9, MPO, elastase and lacioferrin, as indicated

The effects of APPA on degranulation was examined. Neutrophils from healthy controls were pre-incubated for 30 min with APPA (100 µg/mL) and then primed for 30 min with GM-CSF before stimulating degranulation with fMLP and cytochalasin B. Degranulation of primary granules, as measured by CD63 expression using flow cytometry (Fig. 3a), was decreased in APPA-treated cells (*p* < 0.05). APPA also inhibited the release of key secretory molecules, namely MMP9, elastase, MPO and lactoferrin (Fig. 3b) as assessed by analysis of cell-free supernatants of stimulated cells by western blotting.

**Fig. 4:**
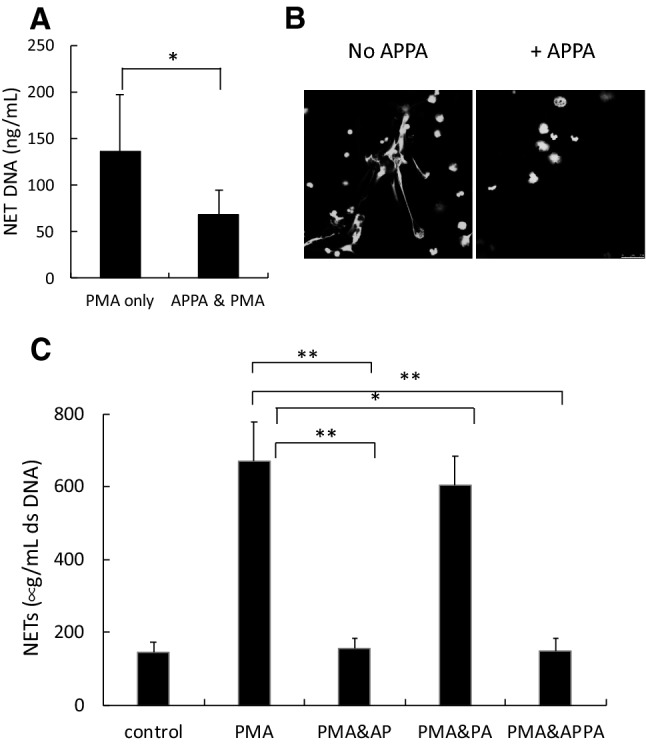
APPA decreases formation of neutrophil extracellular traps (NETs). Neutrophils were treated with PMA for 4 h in the absence and presence of 100 µg/mL APPA. NET formation was measured by DNA release in **a** (*n* = 4, *p = 0.04) and in **b** by microscopy utilizing dual DAPI and neutrophil elastase staining. In **c**, DNA released into NETs was determined after incubation with PMA in the presence of 100 µg/mL APPA, 22 µg/mL AP and 78 µg/mL PA (*n* = 6, **p < 0.05)

The generation of neutrophil extracellular traps (NETs) not only may enhance the trapping and killing of extracellular pathogens (Carmona-Rivera and Kaplan 2016; Branzk et al. 2014; Smith and Kaplan 2015; Knight and Kaplan 2012; Grayson and Kaplan 2016), but also may break immune tolerance by extracellular exposure of autoantigens, thus contributing to autoimmunity (Thieblemont et al. 2016). PMA-stimulated NET production was inhibited by APPA (Fig. 4a,b). This inhibition of NET formation was due to the inhibitory effects of AP (*p* < 0.05) in the APPA mixture, as PA alone had no significant inhibitory effect on PMA-stimulated NET formation (Fig. 4c).

### APPA inhibits key signalling pathways in neutrophils

**Fig. 5:**
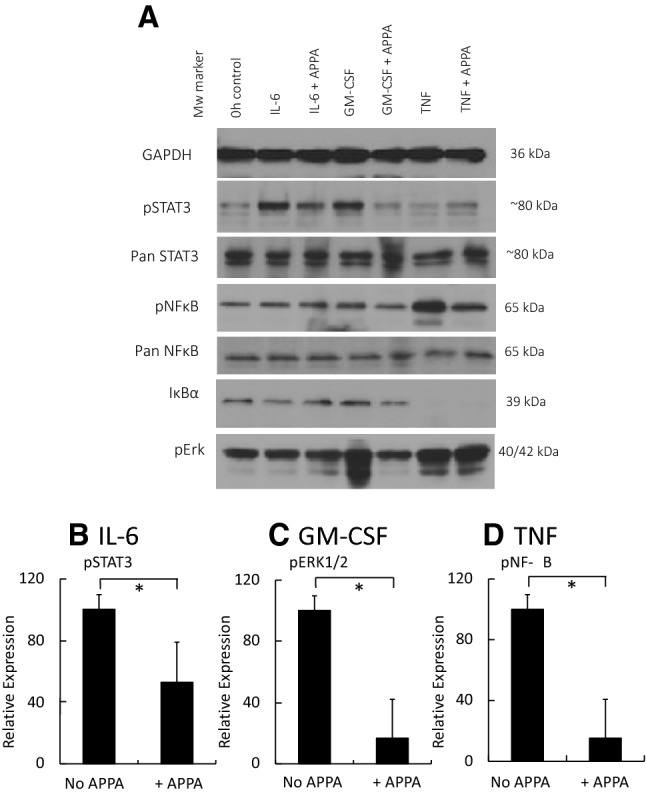
Effects of APPA on activation of cytokine-regulated cell signalling. Neutrophils (5 × 10^6^) were incubated in the absence (UT) or presence of APPA (100 µg/mL) for 10 min. APPA-treated neutrophils were then stimulated for 15 min with either IL-6, GM-CSF or TNFα at the concentrations described in Methods. Western blotting was used to detect activated (phosphorylated) forms of STAT3, NF-κB, IκBα and Erk1/2. **a** Shows typical blot obtained from 3 separate experiments, while **b**–**d** show combined densitometric data (*n* = 3), for IL-6 stimulated STAT3 activation **b**, GM-CSF-stimulated Erk1/2 activation **c** and TNF stimulated NF-κB activation, **d** (*p = 0.03, 0.03 and 0.008, respectively) after normalisation to GAPDH protein levels

Cytokine exposure of neutrophils results in activation of a number of intracellular signalling cascades that trigger events regulating inflammation. These include ERK1/2 and transcription factors such as STAT3 and NF-κB, which are dynamically regulated after exposure of neutrophils to agents such as GM-CSF, IL-6 and TNFα (Mouzaoui et al. 2014; Wright et al. 2014a, b; McDonald et al. 1997). Neutrophils were incubated in the absence (control) or presence of APPA (100 µg/mL) for 10 min before stimulation with GM-CSF, IL-6 or TNFα for 15 min. While IL-6 had only minor effects on neutrophil function, it did activate STAT3; GM-CSF activated STAT3 and pERK phosphorylation; TNFα activated NF-κB (p65 phosphorylation) and enhanced IκBα turnover (Fig. 5a). APPA significantly inhibited IL-6 activation of STAT3 (*p* = 0.03: Fig. 5b), GM-CSF activation of Erk 1/2 (*p* = 0.03: Fig. 5c) and TNFα-mediated activation of NF-κB (*p* = 0.008: Fig. 5d).

### Effects of APPA on neutrophil gene expression

**Fig. 6:**
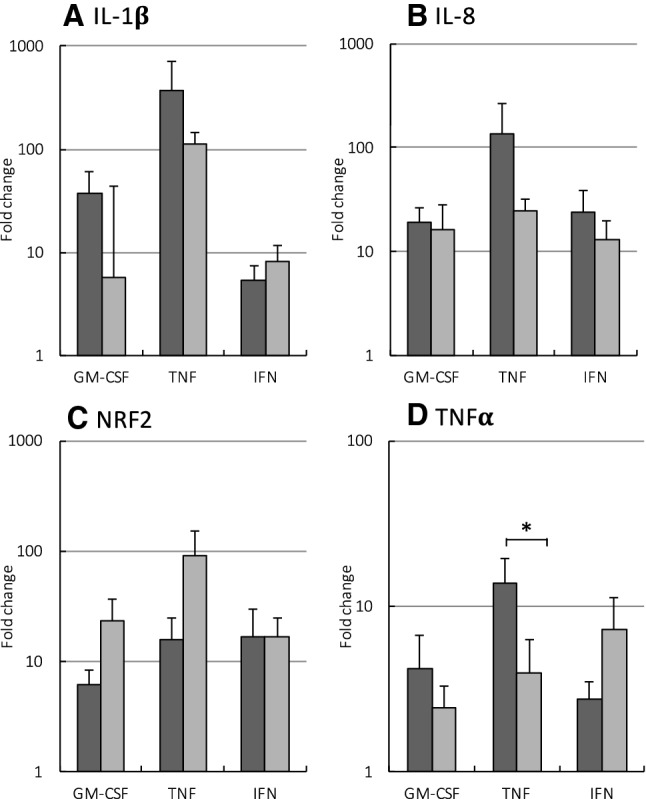
APPA down-regulates TNFα-stimulated gene expression but up-regulates expression of NRF2. Neutrophils (10^7^) from healthy controls were incubated in the absence ( ) or presence ( ) of APPA (100 µg/mL) for 10 min. APPA-treated neutrophils were then stimulated with GM-CSF, IFNγ or TNFα for 1 h. qPCR was used to quantify transcript levels of IL-1β (**a**), IL-8 (**b**), NRF2 (**c**) and TNFα (**d**). Values shown are mean (± SEM), *n* = 6, *p = 0.012

In addition to their ability to prime neutrophils, GM-CSF, TNFα and IFNγ can also rapidly activate neutrophil gene expression. Therefore, we determined if APPA had any effect on the expression of several key neutrophil genes, particularly those regulated by NF-κB. Neutrophils from healthy controls were pre-incubated with APPA (100 µg/mL) for 10 min before stimulation with cytokines (GM-CSF, TNFα or IFNγ) for 1 h. Gene expression was measured using qPCR to quantify transcripts for TNFα, IL-8 and IL-1β. Expression of Nrf2 was also measured as this transcription factor regulates the expression of antioxidant proteins that protect against oxidative stress (Niture et al. 2010; Murakami and Motohashi 2015). Figure 6 shows that cytokine treatment of neutrophils resulted in enhanced expression of IL-1β, (Fig. 6a), IL-8 (Fig. 6b), Nrf2 (Fig. 6c) and TNFα itself (Fig. 6d), and levels of expression of these genes were greater after TNFα treatment than were observed after incubation with either GM-CSF or IFNγ. Pre-treatment of neutrophils with APPA (100 µg/mL) for 30 min resulted in down-regulation of TNFα-activated expression of IL-1β, IL-8 and TNFα, in line with its ability to inhibit NF-κB (Fig. 6). However, APPA enhanced TNFα- and GM-CSF-induced expression of Nrf2 suggesting it is able to induce an anti-oxidative stress response.

**Fig. 7:**
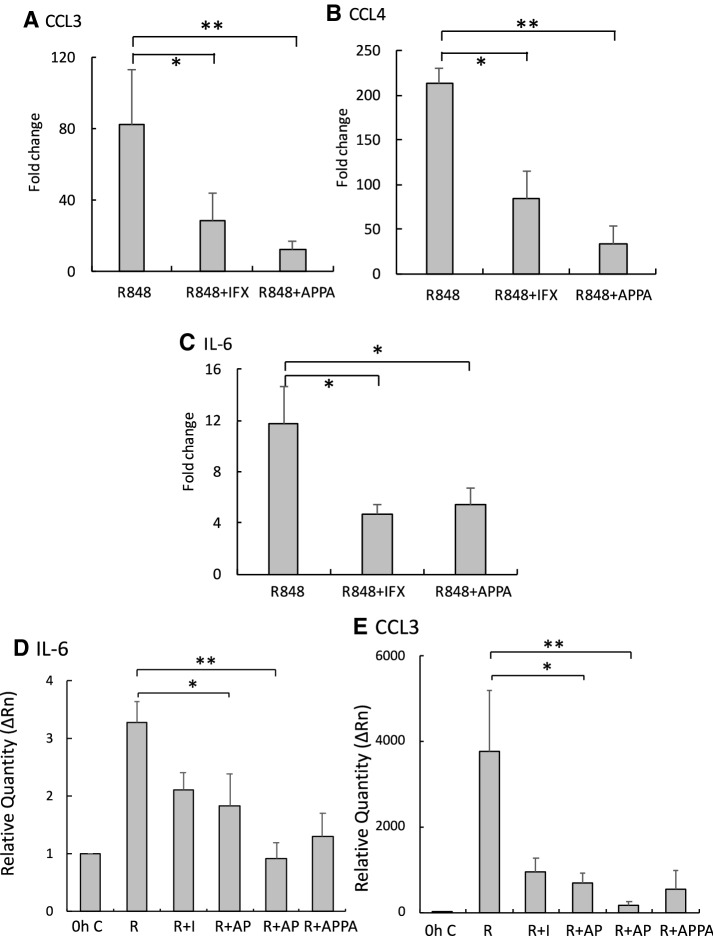
APPA, AP and PA are as effective as infliximab in down-regulating chemokine and cytokine expression. Neutrophils were incubated with 5 µM R848 for 7 h in the absence (R848) or presence of 200 µg/mL infliximab (IFX) or 100 µg/mL APPA. Expression levels of mRNA for CCL3 (**a**), CCL4 (**b**) and IL-6 (**c**) (normalised to GAPDH mRNA levels) were then measured by qPCR. *p < 0.05, **p = 0.01 (*n* = 5). In **d** and **e**, neutrophils were incubated in the presence of R848 (5 µM), APPA (100 µg/mL), Infliximab (IFX, 200 µg/mL), AP (22 µg/mL) and PA (78 µg/mL). Levels of mRNA for IL-6 (in **d**) and CCL3 (in **e**) were measured by qPCR and normalised to GAPDH mRNA levels. Values shown are means ± SEM (*n* = 5). *p < 0.05. **p < 0.01

The recent discovery that neutrophil chromatin can be re-modelled by agents likely to be important in inflammation, to enable transcription of normally silent genes (Zimmermann et al. 2015) has transformed our understanding of the transcriptional repertoire of neutrophils in disease. We, therefore, incubated neutrophils with the chromatin re-modelling agent, R848 for 7 h and measured the effects of APPA on expression of the chemokines, CCL3 and CCL4, and the pro-inflammatory cytokine IL-6. Previous work has shown that endogenous TNFα is important for this R848-induced IL-6 expression (Zimmermann et al. 2015) and so we also incubated R848-treated neutrophils with the neutralising TNFα antibody, infliximab. R848 stimulated the expression of CCL3, CCL4 and IL-6 under these experimental conditions and this expression was significantly decreased in cultures co-incubated with infliximab (Fig. 7a–c), confirming the role of endogenous TNFα in this gene expression. When we measured the effects of the individual componts of APPA on this gene expression, both APPA and AP significantly decreased expression of IL-6 and CCL3, but PA further decreased expression levels to unstimulated, control values (Fig. 7d, e).

## Discussion

APPA and its constituent components, apocynin (AP) and paeonol (PA), have therapeutic effects in several inflammatory settings, explained in part by inhibition of the NF-κB signalling pathway and in part on its ability to scavenge ROS (Glasson and Larkins 2012; Impellizzeri et al. 2011a, b; Riganti et al. 2008). APPA is beneficial in canine OA (Glasson and Larkins 2012; Larkins and King 2017a, b) and may have potential as a therapeutic in human inflammatory conditions. However, its mechanisms of action and possible effects on the immune system must be established before it can be considered as a novel therapeutic for human disease. This report details, for the first time, the in vitro effects of APPA on several key elements of host defence and other relevant functions of human neutrophils. A major challenge in the design of new anti-inflammatory agents is to balance efficacy with safety, particularly ensuring that host defence to infection is protected.

In addition to their well-recognised roles in recognition, uptake and killing of pathogens, human neutrophils can express a variety of important immuno-regulatory molecules such as chemokines, cytokines, growth factors and angiogenic factors (Wright et al. 2014a, b; Jaillon et al. 2013; Cassatella 1995). These molecules regulate the function of other immune- and tissue cells, and their inappropriate release by neutrophils contributes to inflammatory diseases by prolonging or sustaining inflammatory responses (Cassatella 1995). Many neutrophil functions are regulated by rapid activation of kinase cascades that control enzyme activity and/or result in changes in the affinities/surface expression levels of receptors that control opsono-phagocytosis (Cross et al. 2006; Fossati et al. 2002). These functions do not generally require changes in gene expression. However, other neutrophil functions, such as expression of certain chemokines/cytokines, require activation of transcription factors, and perhaps chromatin remodelling, to control de novo gene expression (Wright et al. 2013).

We show here that APPA has little or no effect on essential host-defence neutrophil functions such as receptor expression, uptake and killing of osponised bacteria or chemotaxis. Moreover, APPA did not interfere with cytokine-mediated regulation of these functions under the experimental conditions employed in this study. Some inhibitory effects of high concentrations of APPA were noted (≥ 500 µg/mL), but such concentrations are unlikely to be reached therapeutically (unpublished data, Professor Ian Clark, University of East Anglia). At the lowest concentration used here (10 µg/mL), APPA decreased ROS levels following neutrophil activation by fMLP or PMA. The assay used to detect these oxidants, namely luminol-enhanced chemiluminescence, requires the combined activities of the NADPH oxidase and myeloperoxidase (Edwards 1987), and can be modified experimentally to either measure the production of oxidants or the scavenging effects of anti-oxidants. We show here that the effects of APPA on ROS production are largely via its ability to scavenge oxidants, rather than by preventing their generation. In spite of the fact that the NADPH oxidase is required for the efficient killing of a large spectrum of micro-organisms (Ellson et al. 2006; Zicha et al. 1997), some patients with autosomal recessive chronic granulomatous disease have decreased (but not absent) NADPH oxidase activity, and yet do not always have recurrent infections (Liese et al. 1996). Our experiments described here would support this observation: 10 µg/mL APPA significantly scavenged ROS but did not impair killing of *S. aureus*. An alternative explanation is that APPA did not access the phagolysosome at concentrations sufficient to impair killing. It is also important to note APPA is a scavenger of ROS, rather than an inhibitor of the NADPH oxidase. Therefore, the ion-pumping activities of the NADPH oxidase, necessary for generating optimal protease activity within the phagolysosome (Reeves et al. 2002), will be unaffected by APPA and, hence, microbial killing can still occur, even though ROS may have been quenched. Based on these observations, we conclude that therapeutic doses of APPA are likely to have minimal impact, if at all, on neutrophil-mediated host defence against infection.

ROS production by neutrophils may also activate signalling networks such as MAPKs and NF-κB, to regulate expression of molecules such as IL-8, IL-1β and TNFα (Ndengele et al. 2005). Other groups have shown the importance of the ROS-sensitive MAPKs/NF-κB signalling pathway in the induction of IL-8 in lung epithelial cells (Boots et al. 2012). ERK, STAT3 and NF-κB are suppressed by pre-treatment with PA, indicating that the beneficial therapeutic effect of APPA may be mediated through its antioxidant activity and inhibition of ROS-sensitive inflammatory signalling (Liu et al. 2014). Here, we show that APPA interferes with TNFα-mediated activation of NF-κB and GM-CSF activation of Erk1/2.

It is noteworthy that APPA decreased TNFα-activated expression of IL-8, TNFα itself and IL-1β, although the inhibitory effect on expression of the latter did not reach statistical significance. APPA was also an effective inhibitor of IL-6, CCL3 and CCL4 expression triggered by the TLR8 agonist and chromatin re-modelling agent, R848. This agonist triggers the expression of these genes following chromatin re-modelling via endogenous expression of TNFα, and we show here that APPA (and AP and PA) was as effective as the therapeutic agent, infliximab in the inhibition of this autocrine signalling process. This indicates that APPA could have anti-inflammatory potential, in clinical scenarios in which neutrophils and TNFα signalling play a significant role in pathology, e.g. RA (Wright et al. 2014a, b). APPA also enhanced expression of Nrf2, an anti-inflammatory regulator of anti-oxidant proteins that protect against oxidative stress (Kaspar et al. 2009). Further work is necessary to fully characterise this phenomenon and determine if APPA regulates the expression of other proteins that control cellular responses to stress in inflammatory disease.

## Conclusion

We show that whilst APPA has no significant effects on host defence neutrophil functions such receptor expression, phagocytosis and bacterial killing, it significantly down-regulates TNFα-mediated expression of cytokines and chemokines by neutrophils. This suggests that APPA may have significant anti-inflammatory potential in diseases characterised by dysregulation of cytokine expression or oxidative stress, not only in OA, for which the drug is currently being developed, but also in systemic inflammatory diseases such as RA without suppressing host defence.
